# Intertwined topological phases induced by emergent symmetry protection

**DOI:** 10.1038/s41467-019-10796-8

**Published:** 2019-06-19

**Authors:** Daniel González-Cuadra, Alejandro Bermudez, Przemysław R. Grzybowski, Maciej Lewenstein, Alexandre Dauphin

**Affiliations:** 1 ICFO-Institut de Ciències Fotòniques, Av. Carl Friedrich Gauss 3, 08860 Barcelona, Spain; 2 Departamento de Física Teórica, Universidad Complutense, 28040 Madrid, Spain; 3 Faculty of Physics, Adam Mickiewicz University, Umultowska 85, 61-614 Poznań, Poland; 4 ICREA-Institució Catalana de Recerca i Estudis Avançats, Lluis Companys 23, 08010 Barcelona, Spain

**Keywords:** Topological insulators, Phase transitions and critical phenomena

## Abstract

The dual role played by symmetry in many-body physics manifests itself through two fundamental mechanisms: spontaneous symmetry breaking and topological symmetry protection. These two concepts, ubiquitous in both condensed matter and high energy physics, have been applied successfully in the last decades to unravel a plethora of complex phenomena. Their interplay, however, remains largely unexplored. Here we report how, in the presence of strong correlations, symmetry protection emerges from a set of configurations enforced by another broken symmetry. This mechanism spawns different intertwined topological phases, where topological properties coexist with long-range order. Such a singular interplay gives rise to interesting static and dynamical effects, including interaction-induced topological phase transitions constrained by symmetry breaking, as well as a self-adjusted fractional pumping. This work paves the way for further exploration of exotic topological features in strongly-correlated quantum systems.

## Abstract (web summary)

The still-developing understanding of topologically non-trivial phases of matter has led to new mechanisms for unconventional many-body behaviour. Here the authors present a model where the symmetry needed for a symmetry-protected topological phase only emerges after the formation of long-range order.

## Introduction

The notion of symmetry is paramount to unveil the fundamental laws of Nature, while spontaneous symmetry breaking (SSB) is essential to understand Nature’s different guises^1^. In particular, at long length scales, various phases of matter can be understood by the pattern of SSB and the corresponding local order parameters^2^. Although different SSB patterns tend to compete with one another, a genuine cooperation can also arise in strongly correlated systems with intertwined orders^3^. More recently, topology has been recognized as an exotic driving force shaping the texture of Nature, and leading to phases characterized by topological invariants rather than by local order parameters^4^. It is no longer the breaking of certain symmetries, but actually, their conservation^5^, which gives rise to novel states of matter, the so-called symmetry-protected topological (SPT) phases^6^. In the noninteracting limit, topological insulators and superconductors provide well-understood examples of this paradigm^7^, while current research aims at understanding strong-correlation effects, such as the competition of SPT and SSB phases, due to interactions^8^.

Alternatively, a cooperation between SPT and SSB may allow for intertwined topological phases that simultaneously display a local order parameter and a topological invariant. For integer and fractional Chern insulators, such intertwined orders have been already identified in the literature^9–11^. Nonetheless, in these cases, the topological phases exist in the absence of any protecting symmetry. In more generic situations, the existence of intertwined topological phases will depend on how the symmetry responsible for the SPT phase can be embedded into the broader symmetry-breaking phenomenon. Arguably, the first instance of this situation is the Peierls instability^12^ in polyacetylene, neatly accounted for via the Su–Schrieffer–Heeger (SSH) model at half-filling^13^. Here, the instability leads to a dimerized lattice distortion and a bond-order wave (BOW), where electrons are distributed in an alternating sequence of bonding and antibonding orbitals. A closer inspection shows that inversion symmetry is never broken in such a SSB pattern, which leads to a topological quantization of the electronic polarization^14^, and is ultimately responsible for the protection of the SPT phase.

**Fig. 1:**
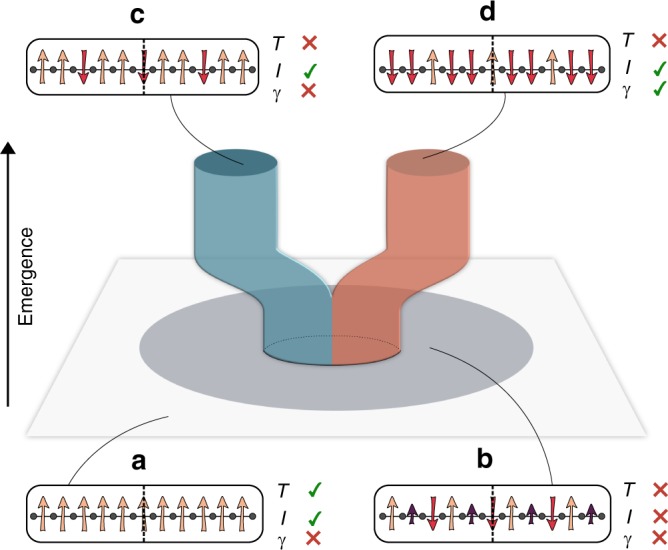
Emergent symmetry protection: we represent qualitatively a ground-state manifold, where different quantum phases are characterized by their symmetry and topological properties, and use spin patterns on the bonds of a 1D lattice to exemplify the different configurations. (**a**) Ground state satisfying both translation (*T*) and inversion (*I*) symmetry, but lacking any nonzero topological invariant (*γ*). The spontaneous breaking of translation symmetry results in a phase with a three-site unit cell, represented in (**b**) with different arrows accounting for the three possible magnetizations, which may not respect the inversion symmetry, lacking a nonzero topological invariant. Remarkably, such inversion symmetry can emerge from all the possible configurations constrained by the SSB pattern, leading to the low-energy sectors depicted in (**c**, **d**). Note that these two phases are not only distinguished by the SSB pattern, but also, and more importantly, by topology. Accordingly, whereas (**c**) is topologically trivial, (**d**) presents both a local order parameter and a nonzero topological invariant, and thus corresponds to an intertwined topological phase where the protecting symmetry emerges

In this work, we study a hitherto unknown possibility: the occurrence of an intertwined topological phase when the SSB pattern does not generally imply the existence of a protecting inversion symmetry (Fig. 1). Instead, this protecting symmetry emerges from a larger set of configurations allowed by the SSB, such that its interplay with topology and strong correlations endows the system with very interesting, yet mostly unexplored, static and dynamical behavior. We demonstrate this topological mechanism in the ${\Bbb Z}_2$-Bose–Hubbard model^15,16^, a microscopic lattice model that displays strongly correlated intertwined topological phases at various fractional fillings. At one-third and two-third filling, and for sufficiently strong interactions, we find a period-3 BOW with a threefold degenerate ground state that displays a nonzero topological invariant: the total Berry phase. We show that inversion symmetry emerges from the larger SSB landscape of a bosonic Peierls’ mechanism, protecting the intertwined topological BOW, and making it fundamentally different from other non-topological BOWs. We unveil a rich phase diagram with first- and second-order quantum-phase transitions caused by the interplay of this emergent symmetry, topology, and strong correlations. We also identify a dynamical manifestation of the underlying topology that is genuinely rooted in strong correlations and the interplay of the emergent and symmetry-broken symmetries: a self-adjusted fractional pump. As discussed by Thouless et al.^17,18^, the quantization of adiabatic charge transport in weakly interacting insulators uncovers a profound connection to higher-dimensional topological phases, as recently exploited in cold-atom experiments^19,20^. Strong interactions can lead to fractional pumped charges^21,22^, showing a clear reminiscence to the fractional quantum Hall effect (FQHE)^23–26^. We show that, following a dynamical modulation of the interactions in the ${\Bbb Z}_2$-Bose–Hubbard model, the system self-adjusts within the landscape of SSB sectors, allowing for a cyclic path that displays a fractional pumped charge 1/3, such that the correlated intertwined topological phase has no free-particle counterpart.

## Results

### ${\Bbb Z}_2$-Bose–Hubbard model

We consider a 1D system of interacting bosons coupled to a dynamical ${\Bbb Z}_2$ field and described by the lattice Hamiltonian

1 $$\begin{array}{*{20}{l}} H \hfill & = \hfill & { - \mathop {\sum}\limits_i {\left[ {b_i^\dagger (t + \alpha \sigma _{i,i + 1}^z)b_{i + 1} + {\mathrm{H}}{\mathrm{.c}}{\mathrm{.}}} \right]} + \frac{U}{2}\mathop {\sum}\limits_i {n_i} (n_i - 1)} \hfill \\ {} \hfill & {} \hfill & { + \frac{\Delta }{2}\mathop {\sum}\limits_i {\sigma _{i,i + 1}^z} + \beta \mathop {\sum}\limits_i {\sigma _{i,i + 1}^x} ,} \hfill \end{array}$$

where $b_i^\dagger$ is the bosonic creation operator at site *i*, $n_i = b_i^\dagger b_i$ is the number operator, and $\sigma _{i,i + 1}^x,\sigma _{i,i + 1}^z$ are the Pauli matrices associated with the ${\Bbb Z}_2$ fields on the bond (*i*, *i* + 1). The bare bosonic Hamiltonian depends on the hopping strength *t*, and the on-site Hubbard repulsion *U* > 0. Likewise, the ${\Bbb Z}_2$ fields have an energy difference between the local configurations Δ, and a transverse field of strength *β* that is responsible for their quantum fluctuations. The ${\Bbb Z}_2$ fields renormalize the bosonic hopping via *α*. Recent experimental progress^27–29^ suggests the possibility of realizing the ${\Bbb Z}_2$-Bose–Hubbard model experimentally using ultracold atoms in optical lattices.

This model (1) hosts Peierls-type phenomena analogous to the fermionic SSH model^13^, but remarkably, in the absence of a Fermi surface^15^. There are, however, important differences: at half-filling and in the slow-lattice limit relevant for polyacetylene^30^, a Peierls’ instability inevitably occurs for arbitrarily small fermion-lattice couplings^12^. In this limit, the fermionic ground state in one of SSB sectors is adiabatically connected to a free-fermion SPT phase^5^. In contrast, our bosonic Peierls phases allow for a genuinely correlated topological bond-ordered wave (TBOW_1/2_) protected by bond-inversion symmetry, which cannot be adiabatically connected to a free-boson SPT phase^16^. We remark that the symmetry protecting the TBOW_1/2_ is completely fixed from the SSB pattern of the ${\Bbb Z}_2$ fields at any energy scale, and is thus not an emergent symmetry.

We now describe a richer situation at filling *n* = 2/3 (similarly *n* = 1/3). For $\Delta \ll t$ and $\Delta \gg t$ the spins are uniformly polarized in the *z* direction, $\langle \sigma _{i,i + 1}^z\rangle = \sigma _0$, with *σ*_0_ > 0 and *σ*_0_ < 0, respectively. For intermediate values, a Peierls-type SSB leads to a trimerization of the ${\Bbb Z}_2$ fields, namely a periodic repetition of a three-site unit cell, with bonds characterized by arbitrary expectation values $\langle \sigma _{1,2}^z\rangle ,\langle \sigma _{2,3}^z\rangle ,\langle \sigma _{3,4}^z\rangle$. The resulting phase is an insulator, with a gap that increases with the value of the coupling *α* (see Supplementary Note 1 for details). Note that this trimerization still leaves freedom for various bond configurations that do not necessarily imply a protecting symmetry for the bosons (Fig. 1b). One of the main results of our work is to show how, for certain parameter regimes, such a protecting symmetry becomes effective at low energies, whereas higher-energy excitations of the ${\Bbb Z}_2$ fields do not necessarily lead to it. Therefore, the inversion symmetry can be understood as an emergent symmetry that is crucial to protect the intertwined TBOW_2/3_ (Fig. 1d). In the following, we set *α* = 0.5*t* and Δ = 0.85*t*.

**Fig. 2:**
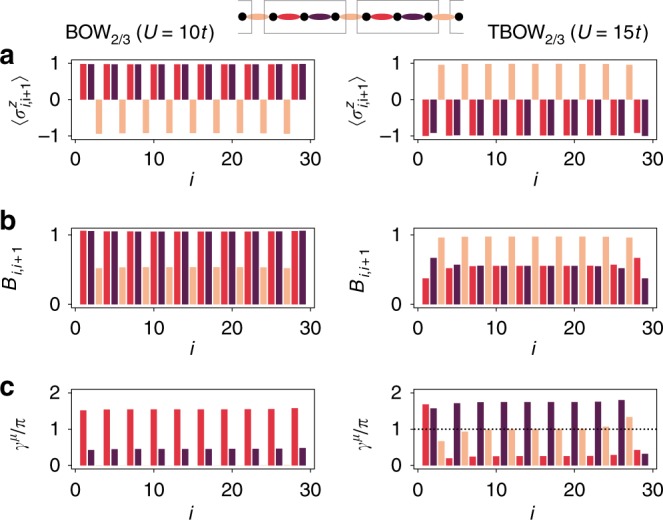
Simultaneous orders in intertwined topological phases: real-space configuration of (**a**) the ${\Bbb Z}_2$ field $\langle \sigma _{i,i + 1}^z\rangle$ and (**b**) the bosonic bond densities $B_{i,i + 1} = \langle b_i^\dagger b_{i + 1}\rangle + {\mathrm{c}}.{\mathrm{c}}.$, using different colors for each element of the unit cell at *β*  = 0.01*t*. Different permutations within the unit cell lead to a threefold quasi-degenerate ground state, each obtained from one another by translating the modulation patterns of the ferrimagnetic and BOW orders. The quasi-degeneracy comes from the finite-size effects, but degeneracy is recovered in the thermodynamic limit. **c**) The local Berry phases *γ*^*μ*^ display a quantized value of 0 (*U* = 10*t*) or *π* (*U* = 15*t*) on the bonds preserving the inversion symmetry of the unit cell, allowing us to distinguish between the trivial and topological BOW phases. We note that the topological BOW phase (right panels) does not have a fermionic analog^48^ in the ground state of the SSH model^32,33^, which instead realizes the trivial BOW (left panels) for energetic reasons

We first study a system of *L* = 30 with sites and open-boundary conditions using DMRG^31^, for *β* = 0.01*t* and different Hubbard interactions *U*. For weak interactions (*U* ⪅ 9t), the ${\Bbb Z}_2$ field is polarized along the same axis (Fig. 1a), and the bosons display a quasi-superfluid behaviour with algebraically decaying off-diagonal correlations. Increasing the interactions leads to a bosonic Peierls transition, whereby translational symmetry is spontaneously broken, leading to a threefold degenerate ground state with ferrimagnetic-type ordering $\langle \sigma _{1,2}^z\rangle = \langle \sigma _{2,3}^z\rangle \, > \, \langle \sigma _{3,4}^z\rangle$, together with a bosonic period-3 BOW that displays inversion symmetry with respect to the central intercell bond (see Fig. 2a, b, left panel). The nature of a similar qSF-BOW phase transition is analyzed in ref. ^16^ for the ${\Bbb Z}_2$-Bose–Hubbard model at half-filling. The BOW phase described here exhibits similar properties to the charge density waves in extended Hubbard models^25,26^, albeit without the need of longer-range interactions. We note that a fermionic counterpart of this phase has been predicted in charge-transfer salts^32,33^. To characterize its topology, we use the local Berry phase $\gamma ^\mu = {\mathrm{i}}{\int}_0^{2\pi } {\mathrm{d}} \theta \langle \psi ^\mu (\theta )|\partial _\theta \psi ^\mu (\theta )\rangle$, where $\left| {\psi _\theta ^\mu } \right\rangle$ is the *μ*th ground state of Hamiltonian (1) with a single bond twisted according to *t* → *t*e^i*θ*^ ^34^. The left panel of Fig. 2c depicts the local Berry phase for one of the ground states, which clearly vanishes on the intercell bonds relevant for the inversion symmetry of Fig. 1. We note that the three possible ground states become degenerate in the thermodynamic limit, which can be characterized by the total Berry phase $\gamma = \mathop {\sum}\nolimits_\mu \gamma ^\mu$. In this limit, the value of *γ*^*μ*^ for the three degenerate states on a fixed bond coincides, up to permutations, with the value of this quantity on the three bonds of the unit cell for each one of the states. Therefore, the sum gives the same value in both cases. For the present BOW_2/3_, we find *γ* = 0, indicating that this phase is topologically trivial.

By further increasing the interactions, a phase with a different SSB pattern $\langle \sigma _{1,2}^z\rangle = \langle \sigma _{2,3}^z\rangle \, < \, \langle \sigma _{3,4}^z\rangle$ arises (right panels Fig. 2a, b). Although the ferrimagnetic and BOW patterns look rather similar to the previous case, the local Berry phase at the intercell bonds is now quantized to *γ*_*μ*_ = *π* (right panel Fig. 2c). Note again that this phase presents three degenerate ground states in the thermodynamic limit, and we find a total Berry phase *γ* = *π*, indicating a nontrivial TBOW_2/3_ phase. This exemplifies the scenario of Fig. 1: from all the trimerized configurations possible a priori, the system chooses one with additional bond-centered inversion symmetry, allowing for a topological crystalline insulator^5^. In combination with the local order parameters (right panel Fig. 2a, b), this shows that the TBOW_2/3_ is an interaction-induced intertwined topological phase, in which, contrary to half-filling^15^, the protecting symmetry is emergent and not fixed a priori by the SSB pattern. The ocurrence of this mechanism is a hallmark of our ${\Bbb Z}_2$-Bose–Hubbard model and does not have an analog in the standard SSH model^32,33^.

### Interaction-induced topological phase transitions

Topological phase transitions delimiting free-fermion SPT phases, and those found due to their competition with SSB phases, are typically continuous second-order phase transitions. In the presence of strong correlations, however, first-order topological phase transitions may arise^35–38^. We now discuss how critical lines of different orders delimit the intertwined TBOW_2/3_ in a strongly interacting region of parameter space, showing that the TBOW_2/3_ cannot be adiabatically connected to a free-boson SPT phase.

**Fig. 3:**
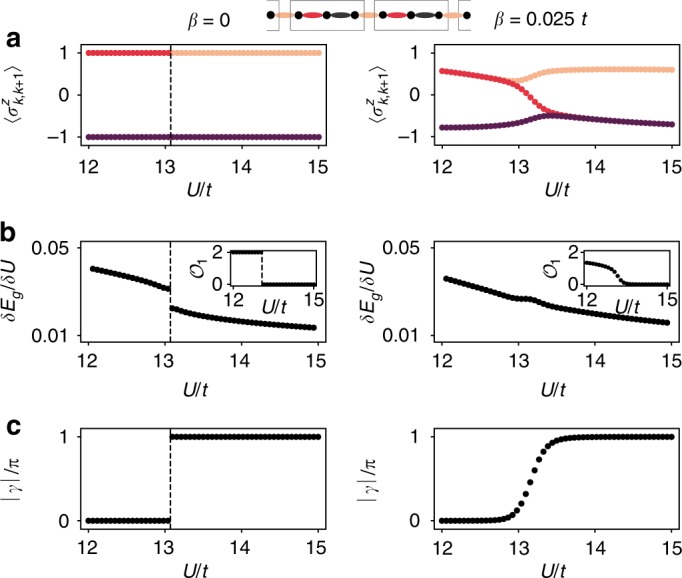
Interaction-induced topological phase transitions: **a** unit-cell fields $\langle \sigma _{k,k + 1}^z\rangle$ as we increase *U*, where *k* ∈ {1, 2, 3} are the three different bonds. The left and right panels correspond to *β* = 0 and *β* = 0.025*t*, respectively. In the first case, there is an abrupt transition between the trivial and topological BOW. In the second case, the transition is continuous, and we find a finite region, where inversion symmetry is broken. **b** First derivative of the ground-state energy per unit cell *E*_g_ through the transition. For *β* = 0, there is a discontinuous jump, signaling a first-order topological phase transition. The inset shows also a jump in the observable ${\cal{O}}_1$. For *β*  = 0.025*t*, both quantities behave smoothly. **c** Total Berry phase, where the same behavior is observed. The results shown are obtained directly in the thermodynamic limit using iMPS (see the “Methods” section)

In the completely adiabatic regime *β* = 0, we observe that the transition between trivial BOW_2/3_ and intertwined TBOW_2/3_ is of first order, using an infinite DMRG algorithm^31^. Figure 3a shows the Ising fields $\langle \sigma _{k,k + 1}^z\rangle$ within the unit cell, as the Hubbard interaction is increased, while keeping *β* fixed. For *β* = 0 (left column), we observe an abrupt transition characterized by a discontinuity in the first derivative of the ground-state energy *δE*_g_/*δU* = (*E*_g_(*U* + Δ*U*) − *E*_g_(*U*))/Δ*U*^36^, signaling a first-order phase transition (Fig. 3b). Introducing the bond observables, ${\cal{O}}_k = \langle E_{\mathrm{g}}|\sigma _{k,k + 1}^z - \sigma _{k + 1,k + 2}^z|Es_{\mathrm{g}}\rangle$ with *k* even or odd, we can characterize the corresponding bond-inversion symmetry within the unit cell. The inset of Fig. 3b shows how ${\cal{O}}_1$ displays a discontinuous jump. The total Berry phase, computed here with the help of the entanglement spectrum^39^, also changes abruptly, as depicted in Fig. 3c. To the best of our knowledge, this is the first topological characterization of a first-order phase transition in an intertwined topological phase.

The situation changes as one departs from the adiabatic regime. Figure 3 (right panel) shows a continuous second-order transition both in *δE*_g_/*δU* and in ${\cal{O}}_1$ for *β* = 0.025*t*. Remarkably, there is a finite region between the trivial and topological BOW phases, where the ${\Bbb Z}_2$ fields have different expectation values, breaking the emergent inversion symmetry within the larger Peierls’ trimerization. These results are in accordance with the behavior of the total Berry phase in Fig. 3c, which shows a nonquantized value in this intermediate asymmetrical region. In fact, the appearance of this region originates from a very interesting interplay between the emergent inversion symmetry and the Peierls SSB phenomenon: a direct continuous transition between the trivial and topological BOWs would require a gap-closing point in the bosonic sector, where every bond had the same expectation value and the BOW would disappear. However, this comes with an energy penalty, since the Peierls’ mechanism favors the formation of a three-site unit cell^15^. Therefore, the system energetically prefers to keep the trimerized unit cell at the expense of breaking the bond-inversion symmetry within the unit cell, and continuously setting the emergent inversion symmetry responsible for the quantized Berry phase *γ* = *π* of Fig. 3c (see Supplementary Note 1 for details). This nontrivial interplay between symmetry protection and symmetry breaking, driven solely by correlations, is another hallmark of our ${\Bbb Z}_2$-Bose–Hubbard model, absent at other fillings or in the fermionic SSH model^32,33^. The intermediate phase could extend up to *β* = 0, although first-order transitions are also possible for low enough values of *β*. An extended numerical analysis would be required to distinguish between these two situations.

**Fig. 4:**
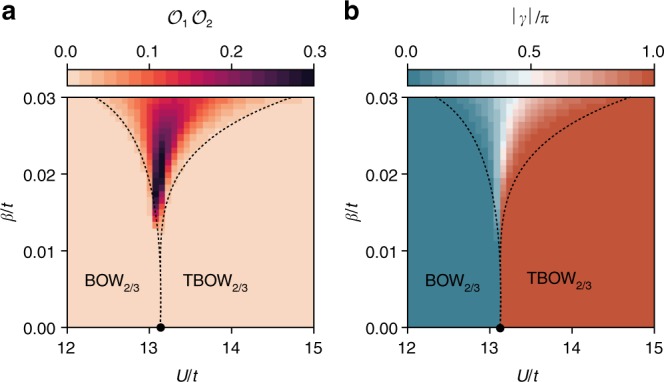
Phase diagram: **a** in the background, we represent the product of observables ${\cal{O}}_1{\cal{O}}_2$, which has a nonzero value only in the intermediate phase, where bond-inversion symmetry is broken. The black dot marks the first-order critical point separating the BOW_2/3_ and TBOW_2/3_ phases at *β* = 0. The dotted lines qualitatively denote the critical lines for *β* > 0. For large values of this parameter, we find an intermediate phase where the bond-inversion symmetry is broken. This phase is separated from the BOW_2/3_ and TBOW_2/3_ phases by continuous transitions. This situation might extend up to *β* = 0, although first-order transitions are also possible for small but not-zero values of *β*. **b** We also present the total Berry phase. The latter has a nonquantized value in the region where the protecting inversion symmetry is broken. The phase diagram is calculated in the thermodynamic limit using iMPS (see the “Methods” section)

Finally, we present the phase diagram as a function of *β* and *U* in Fig. 4a by depicting the product of ${\cal{O}}_1{\cal{O}}_2$: it can only attain a nonzero value if the bond-inversion symmetry within the unit cell is broken (i.e., if the transition occurs continuously via an intermediate nonsymmetric region). Figure 4b shows the phase diagram in terms of the total Berry phase, quantized to 0 and *π* in the regions with inversion symmetry and with nonquantized values in the region where the symmetry is broken.

### Self-adjusted fractional pump

Topology can also become manifest through dynamical effects, such as the quantized transport of charge in electronic systems evolving under cyclic adiabatic modulations: Thouless pumping^17^. This topological pumping lies at the heart of our current understanding of free-fermion SPT phases^7^, and can also be generalized to weakly interacting systems^18^. As advanced in the “Introduction” section, 1D and quasi-1D systems at sufficiently strong interactions can exhibit a fractional pumping^22,40–44^ that cannot be accounted for using noninteracting topological pumping.

**Fig. 5:**
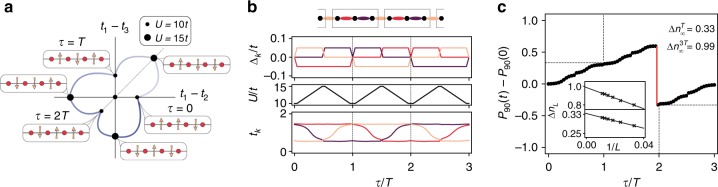
Self-adjusted fractional pumping: **a** the trivial and topological BOW phases are threefold degenerate each. The six different states are represented here as different points on an effective parameter space characterized by the expectation values of the bond fields $t_k = 1 + \alpha /t\langle \sigma _{k,k + 1}^z\rangle$, with *k* ∈ {1, 2, 3}. To define an adiabatic cycle through these different BOWs, the protecting inversion symmetry must be broken at intermediate states in order to enclose the degeneracy point at *t*_1_ = *t*_2_ = *t*_3_. **b** The Peierls mechanism forces the system to break this symmetry spontaneously when interactions are increased, connecting states in the trivial and topological BOW phases (Fig. 3b). In order to select which state from the degenerate manifold the system will transition to, we introduce an external inhomogeneous ${\Bbb Z}_2$ field Δ_*k*_ that is only applied to a subset of bonds within the unit cell. The fields partially break the degeneracy of the BOWs, and restrict the possible adiabatic evolution. A sequential combination of local fields and interaction-driven self-adjustments allows the system to cycle around the degeneracy point in the effective parameter space. Note that the protocol must be repeated three times for the ground state to reach the initial configuration. **c** COM *P*_*L*_(*t*) through the cycle for a finite chain of size *L* = 90 and for *β* = 0.025*t*. The discontinuous jump (red), related to the presence of edge states, allows us to obtain the total charge transported in the bulk during one cycle, Δ*n*_*L*=90_ = 0.92. Inset: finite-size scaling yields a transported fractional charge at *τ*  = *T*, and an integer charge at the end of the adiabatic path (*τ* = 3 *T*)

In this section, we show that adiabatic dynamics traversing through intertwined topological phases allows for a self-adjusted fractional pumping, due to the interplay of the SSB mechanism and other gap-opening perturbations. By introducing guiding fields that only act on a subset of the ${\Bbb Z}_2$ fields, and raising/lowering the Hubbard interactions, the free ${\Bbb Z}_2$ fields self-adjust dynamically during the adiabatic cycle. As a consequence, the bosonic sector traverses a sequence of ground states that are energetically favorable due to the Peierls’ mechanism. In this way, the system self-adjusts along this adiabatic sequence, allowing for an exotic fractional pumping induced by interactions^22,40–44^. The details of this self-adjusted topological pumping are explained in Fig. 5.

For finite systems, the pumped charge can be inferred from the center of mass (COM) $P_L(\tau ) = \frac{1}{L}\mathop {\sum}\nolimits_j {(j - j_0)} \langle \Psi (\tau )|\hat n_j|\Psi (\tau )\rangle$, where *j*_0_ is the center of a chain of size *L*, and |*ψ*(*τ*〉) is the adiabatically evolved state at time *τ*. Figure 5c shows the DMRG results describing how the COM changes along the cycle connecting the BOW_2/3_ and TBOW_2/3_ possible ground states for a finite chain of size *L* = 90. After *τ* = *T*, we observe a COM displacement of ${\mathrm{\Delta }}n_{L = 90}^T = P_{L = 90}(T) - P_{L = 90}(0) = 0.316$, reflecting the fractional charge. To obtain precisely the charge, we perform a finite-size scaling analysis and find ${\mathrm{\Delta }}n_\infty ^T = {\mathrm{lim}}_{L \to \infty }{\mathrm{\Delta }}n_L^T = 1/3$ (inset). At *τ* = 2*T*, the COM displacement reaches a value consistent with 2/3 in the thermodynamic limit. We note that these fractional values are characteristic of a strongly correlated SPT phase with ground-state degeneracy, and cannot be found for any noninteracting topological phase (see Supplementary Note 2 for details). In our present case, the adiabatic path in parameter space can be understood as a dynamical analog of the spatial interpolation between the different ground states, which leads to topological solitons and fractionally quantized charges bound to them^32^. During each period *T*, we interpolate between two such ground states, and a fractional charge is pumped without creating any spatial solitonic profile.

Let us now turn our attention to the discontinuous jump of the pumped charge toward −1/3, as this is related to the presence of many-body edge states for a finite system^45^, and can be used to define a bulk-boundary correspondence for our intertwined TBOW_2/3_. The transported charge across the bulk, $\Delta n_L^{3T}$, can be related to the discontinuous jumps during the cycle^45^, namely ${\mathrm{\Delta }}n_L^{3T} = - \mathop {\sum}\nolimits_i {\mathrm{\Delta }} P_L(\tau _i),$ where ${\mathrm{\Delta }}P_L(\tau _i) = P_L(\tau _i^ + ) - P_L(\tau _i^ - )$ quantify the discontinuities occurring at instants *τ*_*i*_, and $\tau _i^ \pm = \tau _i \pm \varepsilon$ with *ε* → 0. In the thermodynamic limit, it converges to the quantized value of the pumped charge ${\mathrm{\Delta }}n_\infty ^{3T} = {\mathrm{lim}}_{L \to \infty }{\mathrm{\Delta }}n_L^{3T} = 1$ related to the integer Chern number in an extended 2D system^45^. Since these discontinuities depend on the presence of edge states in a finite system, the center-of-mass approach establishes a sort of bulk-boundary correspondence that can be explicitly proven via the adiabatic pumping. Moreover, the COM can be measured in cold-atomic experiments^46^, and it has been used to reveal the topological properties of fermionic and bosonic SPT phases^19,20^.

By estimating the discontinuity, we can extract the transported charge across the bulk during the whole adiabatic evolution that brings the BOW back to itself after *τ* = 3*T*, obtaining a nearly quantized value Δ*n*_*L*=90_ = 0.92. As shown in the inset, a truly quantized charge is recovered in the thermodynamic limit, signaling the topological nature of the system. These results allow us to establish a bulk-boundary correspondence in the pumping process^45^, even though this was not guaranteed a priori, due to the lack of the global symmetries regarding the tenfold classification of topological insulators. In particular, one may understand the edge states of the TBOW_2/3_ as remains of topologically protected conducting edge states of an extended 2D system (see Supplementary Note 2 for details). We note that, even if the topological degeneracy point does not appear in the phase diagram of the model, the quantized transported charge reveals its presence in an effective parameter space, as a nonzero quantized charge can only be obtained when the parameter modulation encircles such a degeneracy point^47^.

## Discussion

We have shown how symmetry protection can emerge through an interplay between symmetry breaking and strong correlations. In the ${\Bbb Z}_2$-Bose–Hubbard model, this mechanism gives rise to an intertwined topological phase for certain fractional fillings. The unique properties of these phases are manifest in the special static and dynamical features discussed in this work. A realistic implementation of the model with cold atoms is suggested by recent experimental results^27,28^. The proposed self-adjusted pumping protocol, in particular, could be used to reveal the topological properties of the system and its fractional nature. Future research directions include the study of topological defects on top of the intertwined topological phases, where localized states with fractional particle number are expected to appear, signaling deeper connections to the physics of the FQHE.

## Methods

### Numerical simulations

The numerical calculations have been performed using a density matrix renormalization group algorithm (DMRG)^31^. For the finite-size calculations, we used a matrix product state (MPS)-based algorithm with bond dimension *D* = 100. To directly access the thermodynamic limit, we used an infinite MPS (iMPS) with a repeating unit cell composed of three sites and *D* = 150. The Hilbert space of the bosons is truncated to a maximum number of bosons per site of *n*_0_ = 2. This is justified for low densities and strong interactions.

## Supplementary information

Supplementary Information

## Data Availability

The data supporting the plots within this paper are available from the authors upon reasonable request. The figures have been produced with Python and adapted with Inkscape and Affinity Designer.

## Code availability

The DMRG calculations have been performed with the tenpy library^31^. The Python scripts used to obtain the data in this paper are available from the authors upon reasonable request.
