# Supplementary material for: Intertwined topological phases induced by emergent symmetry protection
Source: Nat Commun. 2019 Jun 19;10:2694. doi: 10.1038/s41467-019-10796-8 (PMC6584657; doi:10.1038/s41467-019-10796-8)
Supplement: Supplementary file 1 — Supplementary Information [file 41467_2019_10796_MOESM1_ESM.pdf]

Supplemental Information: Intertwined Topological Phases induced by Emergent Symmetry Protection

D. González-Cuadra *et al.*

SUPPLEMENTARY NOTE 1: TOPOLOGICAL PHASE TRANSITIONS

In this part of the Supplemental Information, we provide further details concerning the interaction-induced topological phase transitions discussed in the main text. We recall that the parameters $\Delta = 0.85t$ and $\alpha = 0.5t$ are fixed henceforth.

Gap closure and interaction-induced nature of TBOW$_{2/3}$

The principle of adiabatic correspondence, which states that groundstates that can be adiabatically deformed into each other belong to the same phase of matter, can be used as a primary tool to group groundstates according to a common set of underlying properties. This principle lies at the heart of the modern definition of topological insulators as insulating phases of matter characterized by a topological invariant that cannot be adiabatically connected to the atomic limit (i.e. trivial band insulator). In the context of SPT phases, this principle can be used to delimit the region in parameter space hosting a correlated topological phase, as it must be separated from other states of matter via an abrupt phase transition (i.e. first-order discontinuous transition, or second-order phase transition marked by a gap closure) where the adiabaticity is lost.

In the present context, we have clearly shown in the main text that the TBOW$_{2/3}$ can be connected to the trivial BOW$_{2/3}$ via a discontinuous first-order transition. Additionally, we mentioned that for stronger transverse fields, this transition becomes a continuous second order transition that separates these phases from an intermediate region where inversion symmetry is broken. It is interesting to note that, although the groundstate of the system preserves a trimerized unit cell to satisfy the underlying Peierls mechanism and minimize its energy, the gap of the system does indeed close in this intermediate region, which guarantees that the TBOW$_{2/3}$ is a well-defined phase that cannot be adiabatically connected to the trivial BOW$_{2/3}$. In order to show the occurrence of such a gap closure, we use entanglement spectroscopy in the thermodynamic limit.

Figure 1 shows the scaling of the entanglement entropy $S(\rho_\ell) = -\text{Tr}\{\rho_\ell \log(\rho_\ell)\}$, where ρ_ℓ is the reduced density matrix for a bipartition of the groundstate into two blocks. This entanglement entropy is expressed in terms of the infinite matrix-product state (iMPS) correlation function ξ [1] for different values of the bond dimension D and for different Hubbard interactions U, where we set $\beta = 0.03t$. If the ground state is gapped, this entropy saturates [1]. On the contrary, in a gapless critical point/region, the following scaling relation holds

$$S = \frac{c}{6} \log \xi, \tag{1}$$

for sufficiently large ξ. To capture this saturation or the logarithmic scaling, instead of performing a finite-size scaling, one may conduct a finite-D scaling, which shows how both BOW$_{2/3}$ (Fig. 1(a)) and TBOW$_{2/3}$ are gapped (Fig. 1(c)). In the intermediate symmetry-broken region, we find instead a logarithmic scaling of the entanglement entropy consistent with a conformal charge $c = 1$ (Fig. 1(b)). Accordingly, we can conclude that both phases cannot be adiabatically connected, neither at weak nor at stronger transverse fields. In the regime of small quantum fluctuations (i.e. weak transverse fields), a first-order phase transition takes place. For larger quantum fluctuations (i.e. stronger transverse fields), the gap closes and a a second-order phase transition occurs via a critical region where the emergent inversion symmetry is absent. We note that, when the guiding fields are added in the pumping protocol, the total gap of the system remains open during the cycle, guaranteeing a well-defined adiabatic pumping.

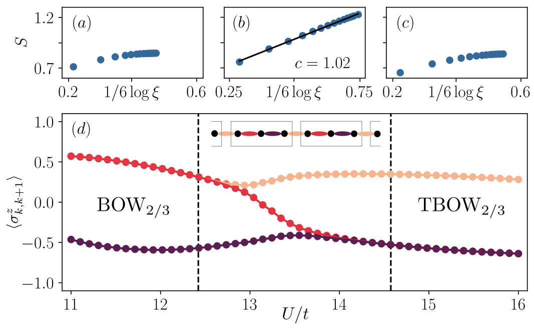

Supplementary Figure 1. **Entropy scaling:** In (a), (b) and (c) we show the entanglement entropy S for an iMPS as a function of the correlation length ξ for a state in the BOW$_{2/3}$, intermediate region and TBOW$_{2/3}$, respectively. In the BOW phases, the entropy saturates, signaling a gapped ground state. In the intermediate region, the scaling is logarithmic. Different points are calculated using different bond dimensions D, and we set $\beta = 0.03t$. (d) Evolution of the $\mathbb{Z}_2$ field on the bonds of the unit cell $\langle \sigma^z_{k,k+1} \rangle$, with $k \in \{1,2,3\}$ in terms of U. The calculations were performed using an iMPS with fixed bond dimension of $D = 150$. The three different phases are separated qualitatively by two dotted lines.

SUPPLEMENTARY NOTE 2: ABSENCE OF FRACTIONALIZATION IN THE EFFECTIVE SINGLE-PARTICLE PUMPING

In this part of the Supplemental Information, we clarify different aspects of the pumping scheme introduced in the main text using an effective non-interacting model. We show that, whereas some qualitative features of the topological pumping in the $\mathbb{Z}_2$ Bose-Hubbard model can be understood through the non-interacting analogue, the very fractional nature lacks a non-interacting simile, and must be consider as a direct manifestation of the strongly-correlated nature of the $\text{TBOW}_{2/3}$.

Bulk-edge correspondence and Chern numbers

Consider the following non-interacting Hamiltonian,

$$\hat{H}_{\text{eff}} = -\sum_i t_i \left(\hat{c}_i^\dagger \hat{c}_{i+1} + \text{H.c.} \right) \quad (2)$$

where $\hat{c}_i$ and $\hat{c}_i^\dagger$ are fermionic operators. $\hat{H}_{\text{eff}}$ is related to the hard-core boson limit of the $\mathbb{Z}_2$BHM, with $U \to \infty$, and a totally adiabatic or classical $\mathbb{Z}_2$ fields ($\beta = 0$) that are treated in a mean-field-like manner. Accordingly, one may consider that the expectation values $\langle \hat{\sigma}^z_{i,i+1} \rangle$ behave as external parameters that can be used to control the effective tunnelling coefficients, $t_i = 1 + \alpha/t \langle \hat{\sigma}^z_{i,i+1} \rangle$.

However, we note that in this simplified effective model, the tunnelings $\{t_i\}$ are free model parameters that can be changed adiabatically at will. In particular, the effective tunnelling strengths are changed following the same path as for the interacting case (Fig. 4(a) in the main text). Note that the expectation values $\langle \hat{\sigma}^z_{i,i+1} \rangle$ extracted from the ground states throughout the self-adjusted many-body pumping are different in general for different unit cells. Being non-interacting, the Hamiltonian (2) can be exactly diagonalized, and the band structure can be obtained at any instant of the adiabatic cycle. This calculation leads to the spectral flow shown in Figure 2(a). Throughout the cycle, the instantaneous energy levels can be arranged into three different bands separated by two gaps that remain finite through the whole adiabatic path. Note also that these spectral bands are connected by two in-gap modes, which correspond to the localized single-particle edge states crossing at some intermediate instant of the cycle. Let us highlight that, in contrast to the adiabatic path of the full interacting model that is composed of three adiabatic cycles, the non-interacting case consists of a single cycle where the effective tunnelings are periodically modulated.

Fig. 2(b) shows the change in time of the center or mass (COM) $P_L(\tau)$, as defined in the main text, for densities $\rho = 1/3$ and $\rho = 2/3$, i.e. when the lowest or the two lowest bands are filled, respectively. We consider a finite chain of size $L = 90$, and show that in both cases a discontinuous jump occurs when the corresponding edge states cross in energy, where the COM changes by 1 or -1. For $\rho = 1/3$, before this instant of time only the left edge state is occupied, while it gets empty and the right edge state gets occupied right after it. This shows the fractionalization of the edge states: if each possesses a particle number of $1/2$, this process changes the COM by $-(-1/2) + (1/2) = 1$. For $\rho = 2/3$, the opposite process takes place. From this discussion it is clear the role that the fractionalization of the edge states plays during the pumping. This pumping appears in both interacting and non-interacting systems and has topological origin. However, as described below, the factional pumping can only appear in interacting systems and is related to the degeneracy of the ground state. In those situations the fractionalization of the pumping is a different effect that goes beyond the fractionalization of the charge of the edge states.

As explained in the main text, we can calculate the transported charge in the bulk from the change in the COM at the discontinuous jumps. We obtain $\Delta n^{1/3}_{L=90} = -0.94$ and $\Delta n^{1/3}_{L=90} = 0.94$. The fact that these are not totally quantized is due to finite-size effects, and they would converge to strictly quantized values in the thermodynamic limit. We thus see that this effective model allows for a net transport of a single quantum of charge accross the bulk, either from the left edge to the right one, or vice versa. Note however that the period T of this single-particle cycle corresponds to the three consecutive cycles $3T$ of the many-body pumping described in Fig. 4(a) in the main text. Therefore, the integer nature of the pumped single-particle charge after one cycle is consistent with the integer value of the pumped many-body charge after three cycles described in the main text.

As noted in the main text, the transported charge in an infinite chain over one period, Δn, gives access to topolog-

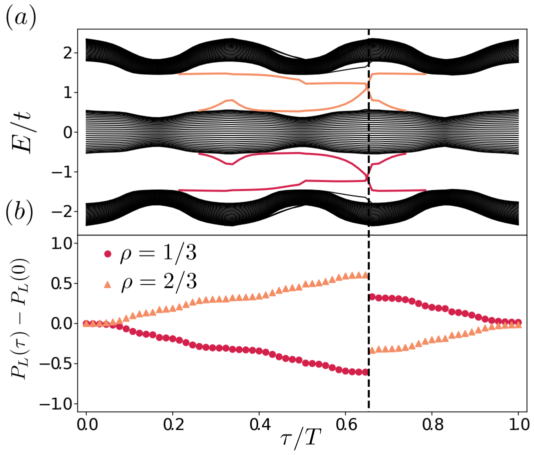

Supplementary Figure 2. **Effective pumping:** (a) spectral flow during the adiabatic cycle. At each time τ we draw the energy levels by diagonalizing the non-interacting effective Hamiltonian (2). We observe three distinct bands that remain open during the cycle. There are two energy levels corresponding to the localized edge states inside each band gap. These states connect the different bands and cannot be adiabatically removed without closing the gap. (b) Change in the center of mass during the cycle for densities $\rho = 1/3$ and $\rho = 2/3$. A discontinuous jump occurs when the edge states in (a) cross in energy.

ical phases in higher dimensions. In this case, the pumped charge can be related to the Chern number of an extended two-dimensional system, where time is taken as a synthetic dimension [2], namely

$$\Delta n = -\sum_n c_n, \quad c_n = \frac{1}{2\pi}\int_0^T \mathrm{d}\tau \int_{\mathrm{BZ}} \mathrm{d}q\, \Omega^n_{q\tau}, \tag{3}$$

where c_n is the Chern number corresponding to the nth band, and the sum is taken over completely filled bands. The Chern number is calculated as a surface integral in the parameter space formed by the time t and momentum q, which has the shape of a torus, and allows to define a Berry curvature

$$\Omega^n_{q\tau} = \mathrm{i}\left(\left\langle \frac{\partial u_n}{\partial q}\middle|\frac{\partial u_n}{\partial \tau}\right\rangle - \left\langle \frac{\partial u_n}{\partial \tau}\middle|\frac{\partial u_n}{\partial q}\right\rangle\right) \tag{4}$$

with $|u_n\rangle$ the n-th Bloch eigenstate. A similar relation to Eq. (3) holds also in the presence of disorder and interactions by introducing twisting fields [3]. We can calculate the associated Chern number for the effective model (2) in the thermodynamic limit using time as a synthetic dimension. Using the efficient numerical method [4], we obtain $c_1 = 1$ and $c_2 = -2$, for the first and second bands, respectively. Using Eq. (3), we obtain the respective charges, $\Delta n^{1/3} = -1$ and $\Delta n^{2/3} = 1$.

This quantized pumping allows to shed light on the origin of the edge states of the trimerized configuration. In contrast to the dimerized half-filling model, the topological origin of the single-particle edge states in this case is not guaranteed a priori due to the lack of chiral/sub-lattice symmetry [5]. However, they can be understood as remains of edge states in the extended two-dimensional system, which are indeed topologically protected even in the absence of chiral symmetry [6]. This is clear from the spectral flow represented in Fig. 2(a), which can be seen as the band structure of a two-dimensional system in a cylindrical geometry [7]. There, the edge states connect the bands separated by a gap, and this means that they cannot disappear under perturbations that do not close the gap. Although this spectral flow can not be computed in the interacting case, we expect the argument to hold based on the quantization of the pumped charge, and the extension of Eq. (3) for many-body systems [3]. Therefore, the observation of the integer pumped charge in the $\mathbb{Z}_2$-Bose-Hubbard model can be used as a bulk-boundary correspondence that clarifies the topological origin of the many-body edge states.

Fragility of the non-interacting fractionalized pumping

A crucial difference between the many-body pumping presented in the main text, and the effective pumping described in this Supplemental Information is that, for the latter, the robust fractionalization of the pumped charge is absent. The main reason behind this is that the ground state of the effective Hamiltonian (2) is not degenerate in the topological phase. Therefore, the adiabatic path can not be decomposed in three independent periodic cycles as in the protocol presented in the main text. As a consequence the transported

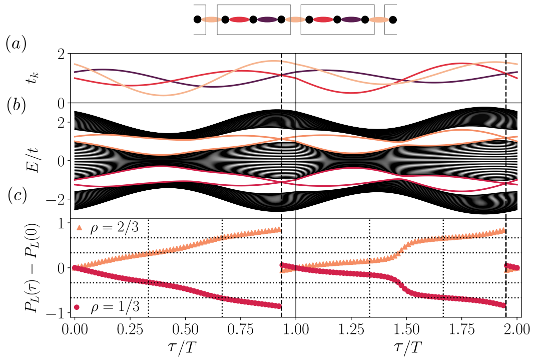

Supplementary Figure 3. **Loss of fractionalization:** (a) Change in the unit-cell tunneling t_k during two pumping cycles which are equal up to an adiabatic deformation. (b) Spectral flow for each cycle. (c) Change in the center of mass during the two cycles. Even if for the first case the transported charge at fractions of the cycle might seem quantized, the second case shows that this is not necessary the case.

charge after a time $T/3$ is not necessarily quantized to $1/3$, only the total charge transported at T is quantized to 1. This can be seen clearly in Figure 3. There, we present two adiabatic pumping cycles, connected by a local deformation. Even if in the first case the transported charge might seem to be fractional quantized for fractions of the period, we observe how this fractionalization is lost in the second deformed cycle. Since the latter is just an adiabatic deformation of the first one, we conclude that the fractional charge is not topologically protected. This is different in the many-body pumping presented in the main text: since the total adiabatic path is decomposed in three closed cycles, any adiabatic deformation would preserve the nature of the pumping, which is robust and quantized to fractional values .

[1] Tagliacozzo, L., de Oliveira, T. R., Iblisdir, S. & Latorre, J. I. Scaling of entanglement support for matrix product states. *Phys. Rev. B* **78**, 024410 (2008).

[2] Xiao, D., Chang, M.-C. & Niu, Q. Berry phase effects on electronic properties. *Rev. Mod. Phys.* **82**, 1959–2007 (2010).

[3] Niu, Q. & Thouless, D. J. Quantised adiabatic charge transport in the presence of substrate disorder and many-body interaction. *Journal of Physics A: Mathematical and General* **17**, 2453–2462 (1984).

[4] Fukui, T., Hatsugai, Y. & Suzuki, H. Chern numbers in discretized brillouin zone: Efficient method of computing (spin) hall conductances. *Journal of the Physical Society of Japan* **74**, 1674–1677 (2005).

[5] Ryu, S. & Hatsugai, Y. Topological origin of zero-energy edge states in particle-hole symmetric systems. *Phys. Rev. Lett.* **89**, 077002 (2002).

[6] Hatsugai, Y. Chern number and edge states in the integer quantum hall effect. *Phys. Rev. Lett.* **71**, 3697–3700 (1993).

[7] Kraus, Y. E., Lahini, Y., Ringel, Z., Verbin, M. & Zilberberg, O. Topological states and adiabatic pumping in quasicrystals. *Phys. Rev. Lett.* **109**, 106402 (2012).
